# Endoscopic ischemic polypectomy using a large endoclip for Peutz–Jeghers polyps

**DOI:** 10.1055/a-2356-7520

**Published:** 2024-07-26

**Authors:** Takashi Taida, Yuki Ohta, Keiko Saito, Kenichiro Okimoto, Tomoaki Matsumura, Jun Kato, Naoya Kato

**Affiliations:** 1 Gastroenterology, Graduate School of Medicine, Chiba University, Chiba, Japan; 2 92154 Endoscopic Center, Chiba University Hospital, Chiba, Japan

Hamartomatous polyps seen in Peutz–Jeghers syndrome are called Peutz–Jeghers polyps (PJPs), and PJPs 15 mm or larger in size may cause intestinal intussusception that requires surgical treatment. The usefulness of balloon-assisted enteroscopy has been reported for PJPs in the small intestine 1 . Although endoscopic resection was the conventional treatment for PJP, endoscopic ischemic polypectomy (EIP), namely the strangulation of the polyps using endoclips without resection, is now preferred for PJPs because of the need to treat many PJPs at one endoscopic session 2 . For polyps with large stalks, conventional clips may not provide sufficient ischemia even with repeated clipping, resulting in the need for snare ischemia 3 . However, the snare technique takes a longer time and is challenging to perform because of the difficulty in obtaining adequate endoscopic images of large polyps and the limited maneuverability of the endoscope 2 . Moreover, if ligation with a snare fails, the snare cannot be reopened and must be discarded, representing a treatment failure. Recently, the SureClip (Micro-Tech) has been developed as a novel endoclip that rotates smoothly and can be reopened after grasping tissue. Although its usefulness in endoscopic treatment has been reported 4 5 , the utility of this device in EIP for PJPs is unclear. The SureClip has available a longer clip width (16 mm) than conventional clips and allows reopening and repositioning, making it easier to achieve strong clamping of the polyps. We report on EIP for PJPs, using the longer clip ( Video 1 ).

Endoscopic ischemic polypectomy (EIP) for Peutz–Jeghers polyps (PJPs) using the longer-width SureClip.Video 1

A 51-year-old man had been diagnosed with Peutz–Jeghers syndrome and treated for PJPs previously. A follow-up computed tomography revealed polyps larger than 15 mm in the small intestine for which treatment by balloon-assisted enteroscopy was required ( Fig. 1 ). Since numerous PJPs were observed in the jejunum, EIP using the clip with the longer opening was performed for polyps bigger than 30 mm. If a polyp with a thick stalk could not be adequately clamped, it was possible to reopen the clip and clamp the stalk appropriately ( Fig. 2 ). Underwater observation revealed floating polyps, and EIP was completed by clamping the stalks of the large polyps using the longer clip to ensure discoloration ( Fig. 3 ) as a sign of adequate ischemia. After treatment, no complications such as bleeding or bowel obstruction were observed.

**Fig. 1:**
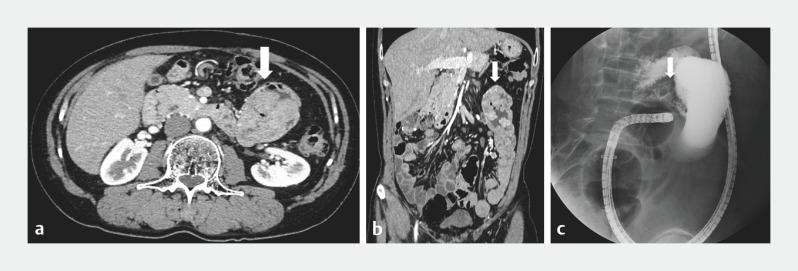
Peutz–Jeghers polyps (PJPs) in a 51-year-old man previously treated for Peutz–Jeghers syndrome. **a, b** Abdominal computed tomography (CT) showed many PJPs (arrows) in the jejunum ( **a** , axial view; **b** , coronal view). **c** Abdominal x-ray showed many polyps (arrow) in the jejunum and dilatation of the intestine proximal to the polyps.

**Fig. 2:**
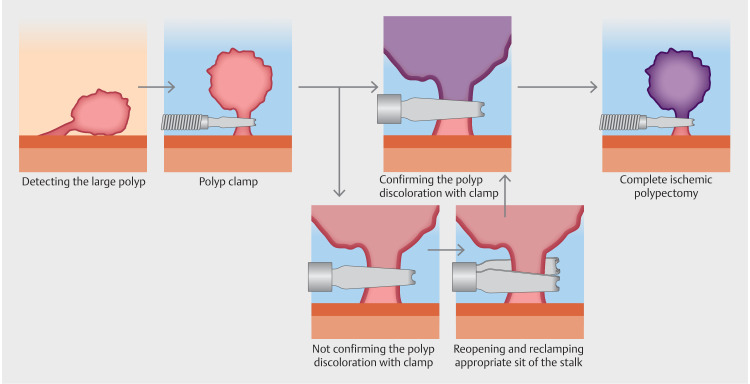
Endoscopic ischemic polypectomy (EIP) using the reopenable clip. The polyp is clamped. If polyp discoloration is confirmed, then EIP is completed. If discoloration cannot be confirmed, then the clip can be reopened and clamping done at another site on the stalk.

**Fig. 3:**
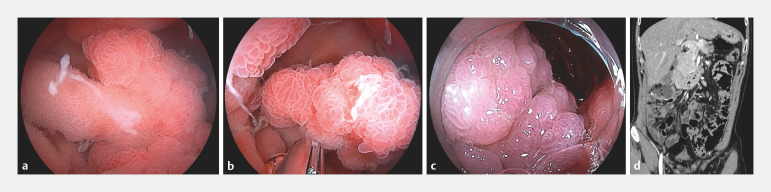
Endoscopic ischemic polypectomy (EIP) for a Peutz–Jeghers polyp (PJP). **a** PJP with a thick stalk floating under water immersion. **b** The polyp stalk is clipped using the longer-width SureClip. **c** Discoloration of the PJP. **d** Abdominal computed tomography shows the decrease in PJPs in the jejunum after EIP.

Because patients with Peutz–Jeghers syndrome usually have multiple large polyps, the reopenable clip with the longer width is effective for secure EIP, and its use reduces procedure time.

Endoscopy_UCTN_Code_TTT_1AO_2AG_3AB

## Notes

Endoscopy E-Videos https://eref.thieme.de/e-videos
*E-Videos* is an open access online section of the journal *Endoscopy* , reporting on interesting cases and new techniques in gastroenterological endoscopy. All papers include a high-quality video and are published with a Creative Commons CC-BY license. Endoscopy E-Videos qualify for HINARI discounts and waivers and eligibility is automatically checked during the submission process. We grant 100% waivers to articles whose corresponding authors are based in Group A countries and 50% waivers to those who are based in Group B countries as classified by Research4Life (see: https://www.research4life.org/access/eligibility/ ). This section has its own submission website at https://mc.manuscriptcentral.com/e-videos .
